# Atomically Precise Nanoclusters as Co‐Catalysts for Light‐Activated Microswimmer Motility

**DOI:** 10.1002/smll.202411517

**Published:** 2025-05-16

**Authors:** John Castañeda, Blake Rogers, Ysaris Sosa, Jorge A. Muñoz, Badri Bhattarai, Ashley M. Martinez, M. Lisa Phipps, Demosthenes P. Morales, Matthew N. Montoya Rush, Miguel José Yacaman, Gabriel A. Montaño, John G. Gibbs, Jennifer S. Martinez

**Affiliations:** ^1^ Department of Applied Physics and Materials Science and Center for Materials Interfaces in Research and Applications (¡MIRA!) Northern Arizona University 1900 S Knoles Dr Flagstaff AZ 86011 USA; ^2^ Center for Integrated Nanotechnologies Materials Physics and Applications Division Los Alamos National Laboratory P.O. Box 1663 Los Alamos NM 87545 USA; ^3^ Department of Surgery Washington University in St. Louis School of Medicine 1402 S Grand Blvd St. Louis MO 63104 USA

**Keywords:** atomically precise nanocluster, hybrid microswimmers, light‐activated microswimmers, photocatalytic active matter

## Abstract

Microswimmers are self‐propelled particles that navigate fluid environments, offering significant potential for applications in environmental pollutant decomposition, biosensing, and targeted drug delivery. Their performance relies on engineered catalytic surfaces. Gold nanoclusters (AuNCs), with atomically precise structures, tunable optical properties, and high surface area‐to‐volume ratio, provide a new optimal catalyst for enhancing microswimmer propulsion. Unlike bulk gold or nanoparticles, AuNCs may deliver tunable photocatalytic activity and increased catalytic specificity, making them ideal co‐catalysts for hybrid microswimmers. For the first time, this study combines AuNCs with TiO_2_/Cr_2_O_3_ Janus microswimmers, combining the unique properties of both materials. This hybrid system capitalizes on the tuned optical properties of AuNCs and their role as co‐catalysts with TiO_2_, driving enhanced photocatalytic performance under ultraviolet (UV) excitation. Using motion analysis, it is shown that the AuNC‐microswimmers exhibit significantly greater propulsion and mean squared displacement (MSD) as compared to controls. These findings suggest that the integration of nanoclusters with semiconductor materials enables state of the art, light‐switchable microswimmers. These AuNC‐microswimmer systems may thus offer new opportunities for environmental catalysis and other applications, providing precise control over catalytic and motile behaviors at the microscale.

## Graphical abstract

For the first time, atomically precise gold nanoclusters (AuNCs) are used as motility catalysts for active matter microswimmers. The nanoscale precision and controlled optical activity of AuNCs are leveraged, achieving a nearly fivefold increase in displacement under ultraviolet light compared to prototypical TiO_2_ microswimmers. This study establishes a route to microswimmers with new functionalities, leveraging the breadth of AuNCs attributes.

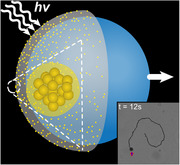

## 1 Introduction

Nature serves as inspiration for materials science and physics alike. Within those natural systems there are potent links between metabolism and motility. Such links enable micron‐sized organisms, such as chemotactic or magnetotactic bacteria, to metabolize chemical nutrients and autonomously move to sense gradients of nutrients or physical forces, as two examples.^[^ ^1^ ^]^

Creation of micronscale materials that mimic Nature's autonomous motility has garnered significant attention both for the study of their physics and for the promise of their future application.^[^ ^2^, ^3^, ^4^ ^]^ These so called “microswimmers” exhibit motility‐induced phase separation and collective behaviors, novel physical phenomena which have broad implications in fields ranging from environmental remediation to medicine.^[^ ^5^, ^6^ ^]^ Several studies have leveraged the unique propulsion mechanisms of microswimmers to perform complex tasks at small scales. For instance, catalytic Janus microswimmers have been demonstrated to navigate biological fluids autonomously and deliver therapeutic agents to disease sites of action with enhanced precision while being minimally invasive.^[^ ^6^ ^]^ Similarly, microswimmers have been explored in environmental remediation, including chemical and biological water purification, where self‐propelled particles degrade organic pollutants through localized catalytic reactions.^[^ ^5^ ^]^ Microswimmers can also be guided remotely using external stimuli, such as magnetic or electric fields, which can enhance their utility in targeted transport and sensing applications in confined environments.^[^ ^7^ ^]^ These advancements emphasize the importance of optimizing microswimmer design and propulsion mechanisms, motivating new strategies to maximize their catalytic efficiency and motion control across diverse applications.

The active control of microswimmer motion has been an active area of research,^[^ ^7^ ^]^ as the fluid drag forces experienced by micron‐sized particles are analogous to the forces experienced by a human swimming in a pool of molasses. Inertia is negligible in this low Reynolds number regime (e.g., water in low flow). Thus, investigators have demonstrated methods to overcome the conservation of momentum that dictates the type of swimming required to achieve motion at these length scales. For example, the use of external electromagnetic fields can drive colloids, e.g. alternating current (AC) magnetic fields, but here we and others are more interested in self‐propulsion akin to chemotaxis. Two primary methods have been established for self‐propulsion: (1) ejection of bubbles, akin to jet‐propulsion, which may be self‐electrophoresis or self‐diffusiophoresis mechanisms or (2) sustained creation of temperature or concentration gradients.^[^ ^8^ ^]^ As the scale decreases, only the former may be possible, but this method requires microswimmers with high catalytic activity and specificity, as well as asymmetry in either the shape or materials properties of the particles themselves.^[^ ^9^ ^]^

Autonomous motion at the microscale is often achieved through catalyzed chemical reactions on a particle's surface. As an example, Janus particles are synthesized with a catalytic material (e.g., bulk gold or platinum on TiO_2_) selectively deposited on one side of the micron‐sized particle.^[^ ^10^ ^]^ Here, the interaction of gold enhances the photocatalytic activity of TiO_2_ by facilitating charge separation and reducing recombination rates of electron‐hole pairs.^[^ ^11^, ^12^ ^]^ When exposed to ultraviolet (UV) light, TiO_2_ generates electron‐hole pairs, with the electrons migrating to the gold.^[^ ^13^, ^14^ ^]^ We and others have demonstrated that migration creates a separation of charges that enhances the decomposition of hydrogen peroxide into oxygen, driving the microswimmer motion.^[^ ^15^, ^16^, ^17^, ^18^, ^19^, ^20^ ^]^ Beyond bulk metal, gold or platinum nanoparticles, as two examples, have been incorporated as catalysts for microswimmers.^[^ ^21^ ^]^ Unfortunately, bulk metal surfaces and nanoparticles may not be selective nor catalytically active enough to enable anticipated applications, particularly as the microswimmer particle size decreases.

Thus, to enable the promised medicinal or environmental applications of microswimmers, which are themselves small particles, the field will need very small and stable onboard catalysts.^[^ ^22^ ^]^ These onboard catalysts must be highly reactive yet enable multiple levels of environmental reactivity and motion control within a single microswimmer, which are well beyond current capabilities. For example, next generation microswimmers capable of combined or sequential response to chemical or physical cues (e.g., different wavelengths of light) may enable more selective toggling and active control of microswimmer trajectories.^[^ ^23^ ^]^ Likewise, the time‐gated synchronization of actuation of all microswimmers may enable the murmuration observed in birds and fish; and the cascade of catalytic reactions, where reactant and products are sequentially consumed by mixtures of microswimmers, may enable microswimmers to be more adaptive to their environment.

Atomically precise metal nanoclusters are multi‐reactive materials tuned in perfect size to serve as the next generation catalysts for microswimmer propulsion, enabling the fundamentally new functionality in active matter as discussed above, and yet, to our knowledge, have not yet been explored as power sources for microswimmers. Nanoclusters have already shown great promise in fields ranging from biomedicine, optics to catalysis. These nanomaterials are comprised of collections of a few atoms of metal (e.g., gold, silver, etc.), ranging in size from the sub‐to a few nanometers.^[^ ^24^ ^]^ Unlike nanoparticles, which have thousands of atoms and support a density of states yielding effects like surface plasmon resonance, ultrasmall nanoclusters support discrete molecule‐like electronic transitions with size or dopant dependent bandgaps.^[^ ^25^ ^]^ We and others have demonstrated that those discrete energy levels promote a strong optical absorbance across the spectrum and, for some nanoclusters, strong photoluminescence ranging from the blue to the near‐IR, which could be highly beneficial for spectrally resolved excitation of associated microswimmers.^[^ ^26^ ^]^ Interestingly, these optically active clusters can be driven into dark states and then actively depopulated in a synchronized way, serving as a potential tool for synchronization of microswimmers.^[^ ^27^ ^]^ Beyond precise control of optical and electrochemical properties, the strong quantum confinement and molecular‐like electronic properties of nanoclusters enable catalysis.^[^ ^28^, ^29^ ^]^ Here, the high surface‐to‐volume ratio and nanostructure catalytic sites have been harnessed for both homogeneous and heterogeneous catalysis, in reactions ranging from hydrogenation, CO_2_ reduction, and water splitting, to name a few.^[^ ^30^ ^]^ This well‐documented history of deposition and stable surface‐mediated catalysis further supports their integration into microswimmers, where their tunable properties can be leveraged to optimize propulsion mechanisms and catalytic performance. Finally, the small size of nanoclusters also enables the first‐principles description of their catalytic mechanism.^[^ ^31^ ^]^

Another attribute of nanoclusters is that their syntheses have been highly refined over the last 20 years. Here, surface ligands template and actively control the solution‐phase synthesis of nanoclusters. These ligands are typically small organic molecules containing metal coordinating moieties such as soft acids, thiols, or phosphines, or larger organic molecules such as DNA, proteins, and synthetic polymers.^[^ ^32^ ^]^ One ubiquitous ligand, used in the controlled synthesis of atomically precise nanoclusters, is glutathione (GSH). Here the reductive decomposition of Au(I)‐GSH polymer yields a series of atomically precise nanoclusters ranging in core diameters from ≈0.8 – 1.2 nm with 10s of atoms each (herein referred to as “Au:SG NCs”, for the as synthesized material, “AuNC”, for the post calcined material).^[^ ^33^, ^34^, ^35^ ^]^ Given the ability to finely tune the size and energy levels of nanoclusters, it is also not surprising that nanoclusters have found application in photocatalysis. For example, Negishi demonstrated that a glutathione protected Au_25_:SG NC could be used as a photocatalytic co‐catalyst for enhanced water splitting on a Ba/La modified TiO_2_ surface.^[^ ^36^ ^]^ Excitingly, these deposited AuNC demonstrated much greater photocatalysis than did larger 8–22 nm Au nanoparticles, and increased stability against poisoning from catalytic products.

**Figure 1:**
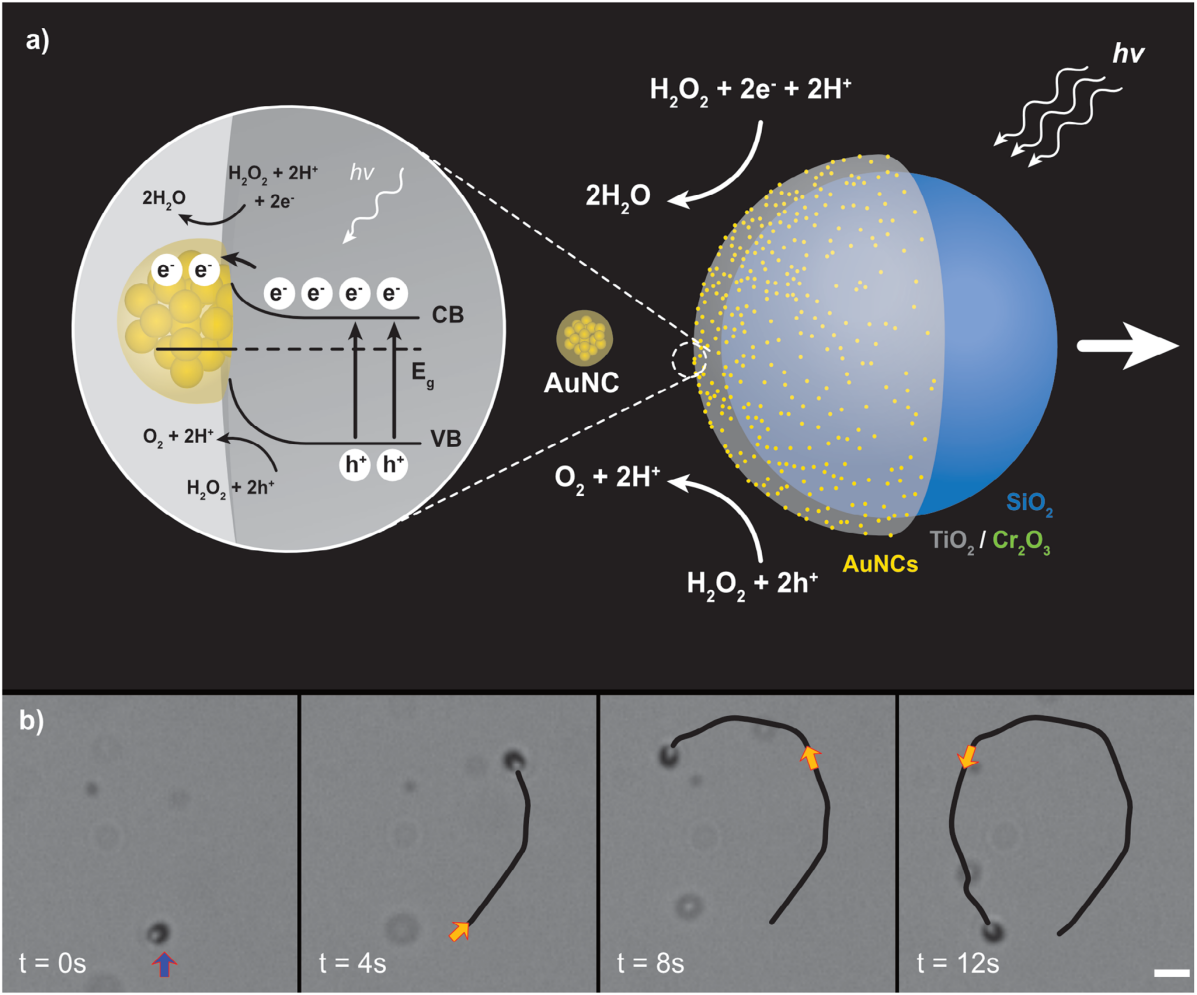
Working principle wherein atomically precise nanoclusters co‐catalyze microswimmer directional motility in aqueous hydrogen peroxide (H_2_O_2_) solution under UV light excitation. a) The electron‐hole (e^−^, h^+^) separation mechanism is likely on the AuNC‐microswimmer surface, driving the oxidation of hydrogen peroxide, as has been demonstrated for TiO_2_ and bulk gold.^[^ ^13^, ^14^ ^]^ The arrows indicate the movement direction and the resultant propulsion force. b) A series of video frames separated by ≈4 s (t = 0 s, 4 s, 8 s, 12 s) capturing the trajectory of a single microswimmer, highlighting its motion path catalyzed by the AuNC‐microswimmer. The colored arrows in the images correspond to the ending point of the previous frame at different stages of the microswimmer's path. The scale bar shown at bottom right is 5 µm for all video frames.

Thus, an opportunity exists to leverage the wealth of cluster synthesis and characterization studies to enable microswimmers with ideal attributes and applications. For the first time we demonstrate here the integration of microswimmers with atomically precise nanoclusters as photocatalysts. In this study, we designed, fabricated and characterized hybrid microswimmers consisting of AuNC‐TiO_2_ (“AuNC‐microswimmers”) capable of photocatalytic redox reactions (**Figure** **1**). First, we synthesized GSH‐stabilized nanoclusters that exhibited a range of atomically precise nanoclusters and their characteristic spectral separation features. Further, we used atomic force microscopy to characterize the distribution of synthesized nanoclusters. Second, we fabricated microswimmers consisting of a 200 nm TiO_2_ layer and an AuNCs layer, separated by a 1 nm Cr_2_O_3_ interlayer. We find that the Cr_2_O_3_ layer enhances the adhesion of Au:SG NCs to the microswimmers and likely plays a critical role in maintaining the structural integrity and catalytic efficiency of the microswimmers.^[^ ^36^ ^]^ Through both qualitative (video) analysis shown in (Figure 1b) and quantitative measure (MSD), we demonstrate that these AuNC‐microswimmers have greatly enhanced light‐driven motion relative to a series of controls. These results provide the first step in the union of two disparate fields, where the precision of atomically precise nanoclusters may be extended to control the motility of microswimmers by using different sized or doped clusters wherein the unique optical and catalytic properties of clusters may synch and tune the microswimmer directionality, without altering the physics of their dynamics due to nanoclusters very small size.

## 2 Results and Discussion

### 2.1 Creation of Hybrid AuNC‐Microswimmers

#### 2.1.1 Titanium Dioxide and Chromic Oxide Janus Particles are Created by Physical Vapor Deposition

**Figure 2:**
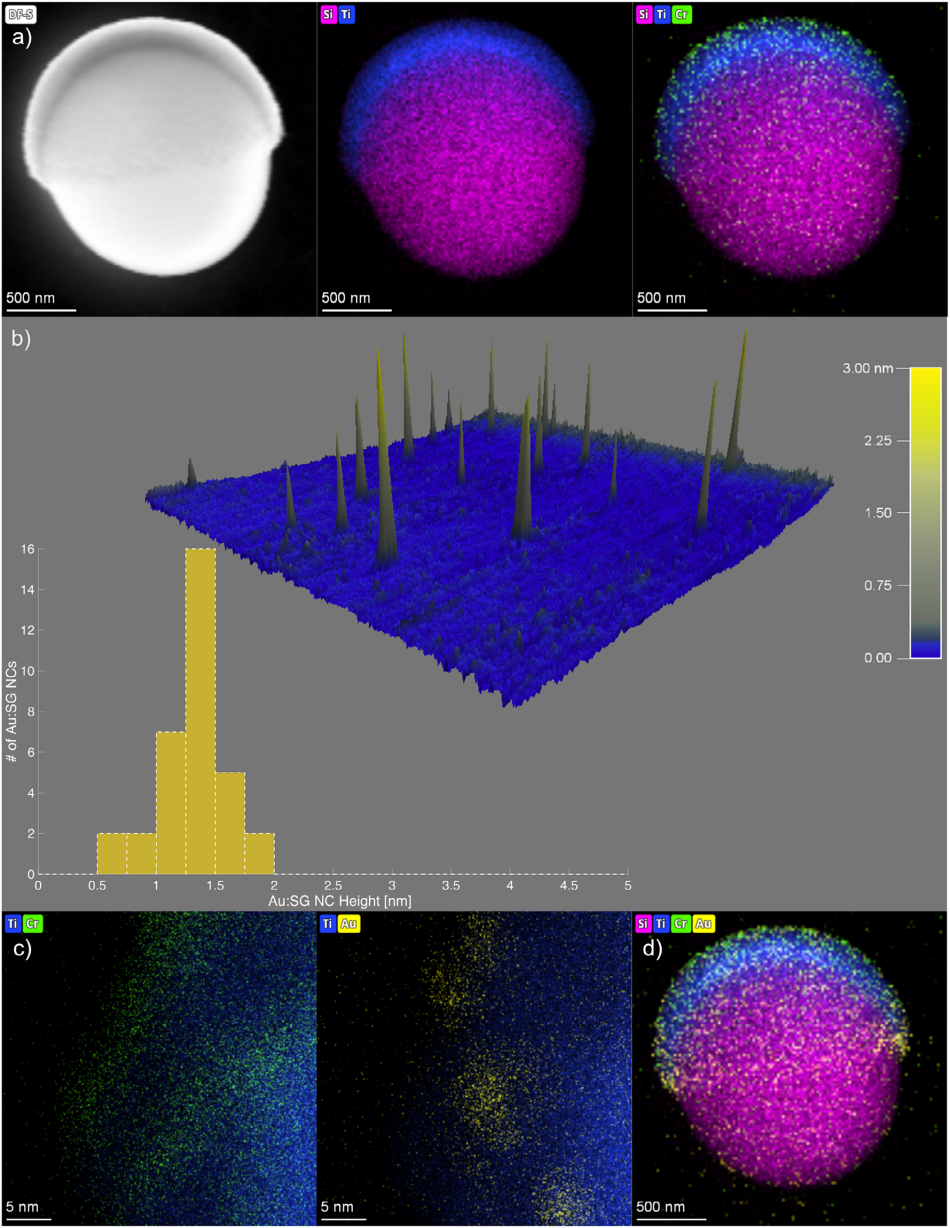
AuNC‐microswimmers were successfully assembled by the deposition of atomically precise nanoclusters (AuNC) onto TiO_2_/Cr_2_O_3_ Janus microparticles. a) Dark field scanning transmission electron microscopy (STEM)/energy dispersive X‐ray (EDX) spectroscopy images demonstrating the microswimmer structure and highlighting the elemental distribution of Si (magenta), Ti (blue), and Cr (green); confirming the successful deposition of the TiO_2_ and Cr_2_O_3_ layers and creation of a Janus structure. b) Atomic force microscopy (AFM) images demonstrating the size of the as synthesized Au:SG nanoclusters. The 3D image depicts the height of the nanoclusters, while the histogram shows the average height distribution of the nanoclusters measured by AFM. NCs range from the <1.0 nm to 2 nm range. The 0 – 5 nm scale is also shown, accurately indicating no materials greater than 2 nm range where measured. c) Magnified STEM/EDX images of the AuNC‐microswimmers after deposition and calcination of Au:SG NCs onto the TiO_2_/Cr_2_O_3_ Janus particles, demonstrating the spatial distribution of AuNC (yellow) on the Ti (blue) and Cr (green) microswimmer surface, in registry. d) AuNC‐microswimmer highlighting the elemental distribution of Si (magenta), Ti (blue), Cr (green), and AuNC (yellow) using STEM/EDX.

We sought to determine whether atomically precise Au:SG NCs could enhance catalysis of TiO_2_ photo‐activated microswimmers (TiO_2_/Cr_2_O_3_ microswimmers). Initial attempts to incorporate Au:SG NCs by drop‐casting them onto the TiO_2_ microspheres were unsuccessful, as the Au:SG NCs did not reliably adhere to the surface and often washed off in aqueous environments. To promote long‐term catalytic durability, we adapted an approach using a thin (1 nm) chromium(III) oxide (Cr_2_O_3_) layer, to improve the adhesion of Au:SG NCs onto titanium oxide surfaces, as demonstrated by Negishi.^[^ ^36^ ^]^ This Cr_2_O_3_ interlayer effectively promotes strong interactions between the TiO_2_ and Au:SG NCs largely confining the clusters to one hemisphere of the particle and reducing detachment or agglomeration.^[^ ^36^ ^]^ Fabrication of TiO_2_/Cr_2_O_3_ Janus particles utilized Physical Vapor Deposition (PVD).^[^ ^37^, ^38^, ^39^, ^40^ ^]^ The deposition began with a monolayer of silica microspheres (2.2 µm in diameter) adhered on a silicon wafer for the deposition of ≈200 nm of titanium at 0° normal incidence in the PVD chamber (see Experimental Section). The created titanium Janus particles were then removed and annealed to convert them into TiO_2_, which provides the photocatalytic properties we aim to enhance.^[^ ^41^, ^42^ ^]^ Following annealing, the monolayer of microspheres was returned to the PVD chamber, and a ≈1 nm Cr_2_O_3_ layer was deposited at 0° normal incidence, ensuring it was confined to the TiO_2_ hemisphere as shown in **Figure** **2**a. The resultant particles present a Janus morphology with bare SiO_2_ and TiO_2_/Cr_2_O_3_ on opposite hemispheres. An earlier system was tested, incorporating a thicker Cr_2_O_3_ layer (≈20 nm, Figure S1, Supporting Information); however, the thicker chromium layer resulted in no appreciable catalysis from either the AuNC‐ nor TiO_2_/Cr_2_O_3_ microswimmers (e.g., only Brownian motion was observed, data not shown). While we note that the thin layers of Cr_2_O_3_ as deposited here by PVD leads to limited Cr concentration, additional interlayers may be explored in the future if applications drive the elimination of this heavy element.

Scanning transmission electron microscopy (STEM) and Energy Dispersive X‐ray (EDX) spectroscopy were used to characterize the microswimmer structure and composition. STEM confirmed that the Janus structure comprised spherical SiO_2_ microspheres ≈2.2 µm in diameter with silicon distribution indicated in purple (Figure 2a). The top hemisphere of the Janus microspheres TiO_2_ layer (blue) was observed along with the Cr_2_O_3_ layer (green) demonstrating successful formation of the Janus structure. The EDX distribution of the titanium layer is relatively thicker than the chromium layer, as is expected for their differences in deposition thickness.

#### 2.1.2 Decorating Titania/Chromium Dioxide Janus Particles with Au:SG NCs

We integrated TiO_2_/Cr_2_O_3_ microswimmers with atomically precise nanoclusters toward creating enhanced photocatalysts. Here the notion is that we may take advantage of nanoclusters ultra small size, offering higher surface area‐to‐volume ratios, as compared to bulk gold or colloidal nanoparticles, and the opportunity for enhanced photocatalysis.^[^ ^13^, ^14^, ^23^, ^43^, ^44^, ^45^ ^]^

The ubiquitous glutathione ligand (GSH) was used to template synthesis of gold nanoclusters.^[^ ^33^, ^34^, ^35^ ^]^ Here the reductive decomposition of Au(I)‐GSH polymer is known to yield a series of atomically precise nanoclusters ranging in core diameters from ≈0.8 – 1.2 nm (many 10s of Au atoms are within each core, yet the diameter does not capture all of the Au atoms nor associated ligands),^[^ ^46^ ^]^ herein referred to as “Au:SG NCs”, for the as synthesized material, and “AuNC”, for the post calcined material. The synthetic methodology is described in the Experimental Section. This material was characterized first by polyacrylamide gel electrophoresis (PAGE), wherein we observed the prototypical nine bands, corresponding to clusters with ≈10‐39 atoms (Figure S2, Supporting Information).^[^ ^34^, ^46^ ^]^ We expect that larger Au NCs (>39 gold atoms) are also present in the larger unresolved bands that can be seen in Polyacrylamide Gel Electrophoresis gel (PAGE). As this effort was proof‐of‐principle in nature, we used the mixture of clusters, and no attempts were made to further purify AuNCs of specific size.

The UV‐Vis spectroscopy of an aqueous dilution of Au:SG NCs revealed absorbance peaks at 325 nm, 375 nm, 450 nm, and 550 nm, consistent with the known UV‐Vis of Au:SG NCs (Figure S2, Supporting Information).^[^ ^34^, ^47^, ^48^ ^]^ Importantly, the spectra do not exhibit a gold surface plasmon resonance, which would have been indicative of gold nanoparticles. This observation was further corroborated by characterization of the Au:SG NCs by atomic force microscopy (AFM). AFM samples were prepared by drop cast or spin coating of the Au:SG NC rehydrated solution on atomically flat mica at concentrations that rendered largely individual nanoclusters at a density capable of visualizing enough NCs for statistical size distribution analysis. AC‐mode AFM was used to analyze Au:SG NCs prior to calcining by measuring the Z‐height of NC's deposited on mica (Figure 2b). We observed that spin coating produced mica surfaces with more dispersed clusters. Au:SG NCs were found to have an average height between 0.5 and 2 nm roughly consistent with the expected size distribution of clusters with monolayer ligands. Figure S3 (Supporting Information) provides the analysis of three separate scans that constitute the histogram analysis provided in Figure 2b. We note that Z‐height analysis, and not width, is the most effective means of analyzing small nanoparticles.^[^ ^49^ ^]^ For example, the cone‐shaped appearance of individual nanoclusters visible in the 3D rendered image of Figure 2b are reflective of “tip broadening” due to the smaller size of the nanoclusters relative to the AFM probe tip radius (≈7 nm). Consistent with the UV‐Vis, we observe no nanoparticle‐sized structures via AFM. Combined, these results confirm the successful synthesis of a mixture of Au:SG NCs with a size distribution consistent with those shown within the literature.^[^ ^46^ ^]^ Excitingly, this demonstration suggests that AFM may be an alternative method to analyze Au:SG NCs alone or in hybrid materials.

**Figure 3:**
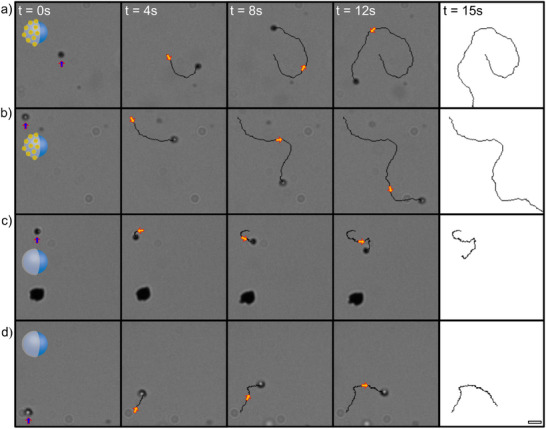
AuNC‐microswimmers enhance the light activated motility and directed trajectories of microswimmers as compared to identical control‐microswimmers lacking the AuNC. Series of video frames separated by ≈4 s, from optical microscopy, capturing the trajectories of microswimmers over 15 s, highlighting the motion catalyzed by their redox active surfaces. a,b) Trajectories of individual AuNC‐microswimmers (AuNC/TiO_2_/Cr_2_O_3_) indicating significant displacement and motion paths due to the enhanced co‐catalytic activity provided by the AuNC. c,d) Control‐microswimmers with TiO_2_/Cr_2_O_3_, but no AuNC, demonstrating much shorter displacement over the same period. The colored arrows in the images mark different stages of the microswimmers' paths (t = 0 s, 4 s, 8 s, and 12 s, with the final trajectory for each at t = 15 s displayed in the rightmost column). The comparison shows the enhanced performance of the AuNC‐microswimmers in terms of displacement. The environmental conditions and scale bar for all videos are the same. H_2_O_2_ concentration of 5% and UV light excitation at λ = 365 nm with scale bar shown at bottom right of 5 µm for all video frames.

**Figure 4:**
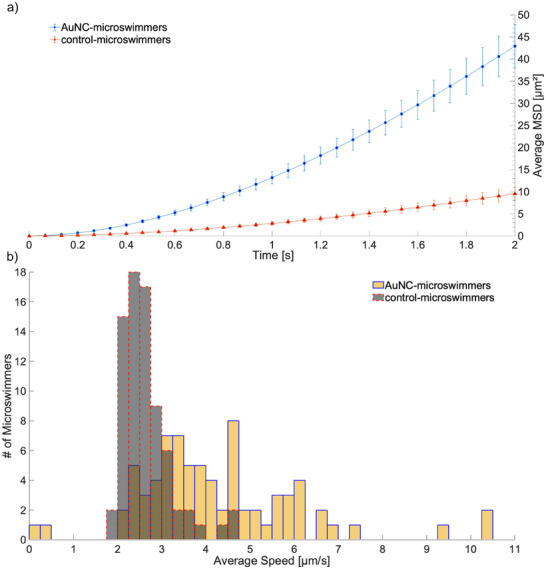
The enhanced light activated motility of AuNC‐microswimmers results in a ≈4.5 fold increase in MSD scaling and a shift of the particle population toward higher speeds, as compared to control‐microswimmers without AuNC. a) The MSD for the AuNC‐microswimmers (blue, *n* = 76) and the control TiO_2_/Cr_2_O_3_ microswimmers (red, *n* = 75). The data demonstrates a significant enhancement in MSD for the AuNC‐microswimmers, indicating improved motility and catalytic activity due to addition of AuNC. The mean velocity and diffusion coefficients were approximated using Equation 4 for particles undergoing ballistic motion. The AuNC‐microswimmers had a mean velocity of 2.97 ± 0.21 µm s^−1^. While the control TiO_2_/Cr_2_O_3_ microswimmers had a mean velocity of 1.42 ± 0.07 µm s^−1^. b) The frequency distribution of the average speeds for the AuNC‐microswimmers (yellow) compared to the control TiO_2_/Cr_2_O_3_ microswimmers (gray). A two‐sided Mann‐Whitney U test indicates a significant difference between the control and AuNC‐ microswimmers (U = 895, *p* < 0.001). Visually the control microswimmers show a clear peak in average speed, centered over a spread in velocity between ≈2 and 4 µm s^−1^, while the AuNC‐microswimmers exhibit a broader distribution and higher average speed distribution, highlighting the enhanced propulsion efficiency and variability introduced by the AuNC.

For adhesion to TiO_2_/Cr_2_O_3_ microswimmers, the undiluted Au:SG NC solution (10 mg Au:SG NC powder rehydrated in 1 mL nanopure water) was drop‐cast onto the microswimmer surface. To ensure proper adhesion and interaction between the Au:SG NC and the TiO_2_/Cr_2_O_3_ surface, the Au:SG NC microswimmers were calcined under vacuum. This process was performed to remove the glutathione (GSH) ligand surrounding the AuNC,^[^ ^36^ ^]^ enabling direct contact between AuNC and the TiO_2_/Cr_2_O_3_ surface. The Cr_2_O_3_ layer played a crucial role, facilitating AuNC adhesion and stabilization of the AuNCs on the TiO_2_ surface, while still being thin enough to enable photocatalysis. STEM and EDX analysis demonstrated that the deposition of the AuNC roughly followed the deposition of Cr_2_O_3_. Where the chromium layer was deposited, so too was the AuNC (Figure 2c, Cr and Au EDX of the same micrograph, in registry). We aimed for an ultrathin chromium adhesion layer, which resulted in a spotty surface. We note that this sparse deposition of the chromium layer may have hindered the ultimate degree of AuNC deposition and thus limited the enhanced catalysis within the AuNC‐microswimmers and that a more uniform adhesion layer may be more beneficial. Further, it was observed that the AuNCs did not deposit uniformly within the Janus hemisphere. STEM images (Figure 2d) revealed that the nanoclusters, in addition to deposition over the Janus hemisphere, also appear to have a substantial deposition within a “ring” or “sideburn” pattern along the boundary between the hemispheres. This irregular distribution likely stems from the drop‐casting process, where closely packed monolayers of microspheres on the silicon wafer caused the Au:SG NCs solution to settle and dry along the inter‐particle regions. Additionally, EDX mapping in Figure 2 directly confirms the attachment of AuNCs, and although the observed quantity appears relatively low, the significant enhancement in microswimmer motility in **Figures** **3** and **4** suggests strong co‐catalytic efficiency of the attached clusters.

### 2.2 Hybrid Microswimmers are Propelled with AuNC Co‐Catalysts

#### 2.2.1 AuNC‐Microswimmer Motion

By incorporating Au:SG NCs into our microswimmer system, we sought to leverage the known catalytic enhancement produced by TiO_2_ and gold heterojunctions.^[^ ^41^, ^42^ ^]^ In general, for TiO_2_ microswimmers, the general mechanism for catalysis in H_2_O_2_ is:

(1) $$2H_{2}O_{2}\to 2H_{2}O+O_{2}$$

which proceeds via electron and hole‐based partial reactions.^[^ ^11^, ^12^, ^19^ ^]^ The resulting concentration gradients drive phoretic flows over the microswimmers, contributing to their self‐propulsion.

While our studies were not mechanistic, we reasoned that the presence of AuNCs as co‐catalysts may enhance charge separation by increasing the distance between photogenerated electron‐hole pairs, reducing charge recombination, and thereby improving catalytic activity of the TiO_2_/Cr_2_O_3_ microswimmers, which was our focus.^[^ ^13^, ^14^, ^23^, ^43^, ^44^, ^45^ ^]^ Thus, our study aimed to determine whether adhering AuNCs to TiO_2_ microswimmers via a Cr_2_O_3_ monolayer could enhance the motility of photo‐activated microswimmers. In our system, Cr_2_O_3_ serves as a critical adhesion layer, ensuring the persistence of the AuNCs on the TiO_2_ microswimmer surface. Together, these components should work synergistically to improve the motility and stability of the microswimmers under UV light activation.

For clarity, “AuNC‐microswimmers” are TiO_2_/Cr_2_O_3_ microswimmers with deposited AuNC, whereas the “control‐microswimmers” are TiO_2_/Cr_2_O_3_ microswimmers where AuNC were omitted. Once synthesized, these AuNCs‐microswimmers were resuspended in nanopure water with a fixed 5% H_2_O_2_ concentration for motility measurements (see Experimental Section). AuNC‐microswimmers with hydrogen peroxide and UV excitation exhibited non‐Brownian motion and significantly more pronounced motion trajectories relative to the control TiO_2_/Cr_2_O_3_ microswimmers (Figure 3a‐b). The motion trajectory for observed AuNCs‐microswimmers demonstrate enhanced translational or rotational motion about their starting position with the distance from the starting point increasing as a function of time. In contrast, the motility of the control TiO_2_/Cr_2_O_3_ microswimmers demonstrated similar motion, though significantly reduced relative to AuNCs‐microswimmers shown in (Figure 3c‐d). The apparent randomness in AuNC‐microswimmer directionality demonstrated for all videos in Figure 3a‐d can be attributed to several factors. First, for many active systems the MSD exhibits two primary time frames: an initial short ballistic time frame followed by a longer diffusive mode time frame.^[^ ^50^ ^]^ In the ballistic time frame, motion is dominated by self‐propulsion, where microswimmers begin to move freely with minimal external perturbations after receiving an initial photocatalytic impulse. This phase is characterized by a quadratic dependence of MSD on time (MSD ≈ t^2^), indicative of directed motion. As time increases, neighboring collisions and rotational diffusion become more prominent, transitioning the system into a diffusive time frame where motion follows a linear dependence on time (MSD ≈ t), with microswimmers exhibiting more random walk behavior. We quantitatively assess the ballistic contributions further in the next section. Second, in addition to particle collisions, we also expect that there could be additional influences that may affect the particle's motion. For example, the non‐uniform coating of AuNCs on the TiO_2_ microswimmers likely introduces asymmetric catalytic activity, resulting in varying forces that drive changes in direction and vary from swimmer to swimmer. Additionally, the closed cell environment places microswimmers in close proximity, where interactions between neighboring particles such as hydrodynamic forces or localized chemical gradients further disrupt consistent motion.^[^ ^51^, ^52^, ^53^, ^54^ ^]^ The lower motility for TiO_2_/Cr_2_O_3_ microswimmers, relative to AuNC‐microswimmers, emphasize the critical role of AuNCs in enhancing motility. However, the observed directional changes confirms that environmental constraints and surface effects influence AuNC‐microswimmer behavior over time. These findings highlight the importance of optimizing both the microswimmer design and experimental conditions to achieve controlled motility. Future investigations could explore strategies for achieving more uniform AuNC and/or Cr_2_O_3_ coatings or patterned deposition to reduce randomness and promote consistent directional motion, as well as testing in open systems to mitigate confinement‐induced variability.

#### 2.2.2 Quantitative Measure of AuNC‐Microswimmers Motility

The observed trajectories and increased displacement of AuNC‐microswimmers, relative to all control‐microswimmers motivated us to quantitatively evaluate their motility.

Videos were recorded for the microswimmers (see Supporting Information), and the 2D trajectories of each particle were extracted and tracked for a minimum of 10 s, corresponding to ≈150 frames per particle. These trajectories allowed us to infer the strength of propulsion by determining the MSDs and average speeds. Further, we focused on the MSD at time intervals below two seconds, a timeframe where propulsive forces dominate before neighboring environmental collisions and rotational diffusions, Δ*t* ≪ τ_*r*_, alter movement trajectories at later time points.^[^ ^50^, ^55^, ^56^ ^]^ The experimental diffusion coefficients for particles in two dimensions (*n* = 2) were estimated using Equation 2 to compare them to the theoretical diffusion coefficients derived from the Stokes‐Einstein equation [Equation 3, Boltzmann's constant k_b_, a temperature of (T = 298 K), the viscosity of water (*n* = 0.89 mPa.s), and the microswimmer radius of (r = 1.1 µm)]. Generally, within an experiment diffusion coefficients were comparable, and the calculated differences were within expected ranges, given the microswimmers’ closed environment (contributing near‐surface effects) within the microscope observation cell.^[^ ^51^, ^52^, ^53^, ^54^ ^]^

(2) MSD=2nDt

(3) $$D=\frac{k_{B}T}{6\pi \eta r}\;$$

(4) $$MSD≅\;v^{2}\Delta t^{2}+4D\Delta t$$

Several conditions were measured: TiO_2_/Cr_2_O_3_ Janus microswimmers with and without AuNCs and analysis with and without UV excitation (365 nm) or visible light excitation (562 nm). Varying the excitation wavelength was used to determine motility as a function of absorbance strength for both high and low concentration of AuNCs, which exhibit a significantly decreased absorption in the visible (562 nm) region (Figure S2, Supporting Information).

AuNC‐microswimmers and TiO_2_/Cr_2_O_3_ microswimmers with peroxide but without UV excitation exhibited no propulsion (Figure S4, Supporting Information) as evidenced by the linear trend and minimal change in the MSD across the time intervals. Excitation with 562 nm light also did not result in catalytically driven motility (Figure S5, Supporting Information) indicating that direct photoexcitation at these higher wavelengths does not result in appreciable enhanced motility of the microswimmers, under current design. We found that all non‐excited controls (e.g., dark controls) and those experiments excited in the green demonstrated Brownian motion and the diffusion coefficients validate the absence of propulsion. Fitting Equation 2 to these MSD curves, we calculated the diffusion coefficients to be ≈0.1 µm^2^ s^−1^, based on the time intervals up to 2 s. Comparing this to the theoretical diffusion coefficient of ≈0.223 µm^2^ s^−1^ for microparticles of ≈1.1 µm, derived from the Stokes‐Einstein equation, we note a lower experimental value, consistent with the effects of the closed microscope observation cell. The reduced diffusion rate can be attributed to near‐surface interactions and hydrodynamic constraints, which limit free Brownian motion compared to theoretical conditions in ideal, open systems.

When comparing MSD for the AuNC‐microswimmers and TiO_2_/Cr_2_O_3_ microswimmers, as shown in Figure 4a, our analysis reveals that the MSD scales nearly four‐ to fivefold more for the AuNC‐microswimmers as compared to the control TiO_2_/Cr_2_O_3_ microswimmers. Specifically, the MSDs of the AuNC‐microswimmers increased on average to ≈43 µm^2^ compared to ≈9 µm^2^ for control TiO_2_/Cr_2_O_3_ microswimmers at a lag time of two seconds. We estimated the mean velocity of the active particles undergoing ballistic motion by fitting Equation 4 to the MSD curves. The mean velocity of the particles to be 2.97 ± 0.21 and 1.42 ± 0.07 µm s^−1^ for the AuNC‐microswimmers and the control microswimmers, respectively. This twofold difference corroborates the enhanced motility observed visually, confirming that the presence of AuNCs boosts the microswimmers' movement.

The average speed per particle for each set of microswimmers was also collected to provide an additional statistical comparison between the active and control groups. A two‐sided Mann‐Whitney U test performed on the frequency distribution of the average speeds and a significant difference was observed between the control microswimmer and the AuNC‐microswimmer measurements (U = 895, *p* < 0.001).^[^ ^57^ ^]^ The control TiO_2_/Cr_2_O_3_ microswimmers exhibit a narrow distribution of average speeds ranging from ≈1.75 to 4.75 µm s^−1^, whereas the AuNC‐microswimmers exhibited a wider distribution of speeds, ranging from ≈2 to 11 µm s^−1^ (Figure 4b).

Note that the observed average speeds are greater than the mean velocities calculated from the MSD fit. This is because the frequency distribution of average speeds inflates the observed values by combining contributions from random motion and directed motion into one measure. In contrast, when fitting the MSD data using Equation 4, the active motion is separated from diffusive motion, allowing the velocity to reflect only the directed component. This separation inherently lowers the calculated mean velocities compared to the average speeds derived from the raw data, offering a clearer distinction between active propulsion and random diffusive motion of our microswimmers.

Decreasing the concentration of AuNCs adhered to the microswimmers by ≈6.25‐fold resulted in a corresponding three‐fold reduction in MSD scaling from the higher concentration (Figure S6a, Supporting Information). The histogram in Figure S6b (Supporting Information) shows a broad distribution in speeds observed for the lower concentration of AuNC in the AuNC‐microswimmers and may suggest the cluster suspension was less uniform than anticipated, resulting in some microswimmers that received substantially more, less or little deposited AuNC. We note that experimental deposition methodology from AFM experiments suggests that the method of deposition (e.g., spray coating over drop casting, as done for catalysis) substantially changed the distribution of Au:SG NCs. Thus, these results may indicate potential strategies to increase reactivity of AuNC‐microswimmers generally. As compared to previously reported reactivity of nanoparticles (1000s of atoms) the enhanced motility observed in our AuNC‐microswimmers reflects the nanoscale interactions facilitated by Au:SG NCs. While bulk gold systems rely on Schottky barrier effects for charge separation,^[^ ^14^, ^44^ ^]^ our system likely leverages a higher density of catalytic sites due to the nanoclusters’ atomically precise structure, resulting in a nearly fivefold increase in motility compared to our control microswimmers. Our system's MSD and speed results, compared to both bulk and nanoparticle‐based systems, emphasize the potential of nanoclusters for advanced microswimmer design. Future studies might explore uniform AuNC coatings and hybrid systems to enhance spectral responsiveness and achieve greater control over directionality in diverse catalytic applications.

The broader speed distribution observed for AuNC‐microswimmers contrasts with the more uniform speeds reported for gold nanoparticle or bulk gold systems. A study of TiO_2_ microspheres with embedded Au nanoparticles demonstrated enhanced photocatalytic activity due to the localized surface plasmon resonance (LSPR) effect of Au nanoparticles enhancing visible light absorption and charge separation.^[^ ^43^ ^]^ In comparison, our AuNC‐microswimmers achieve enhanced photocatalytic performance under UV light. Further, in a previous study looking at bulk gold, Au/B‐TiO_2_ Janus micromotors achieved MSD values of ≈10–15 µm^2^ for 2 s time intervals under UV light with 3% hydrogen peroxide,^[^ ^14^ ^]^ whereas our AuNC‐microswimmers reached ≈43 µm^2^ for the same time intervals in 5% hydrogen peroxide. These results suggest that the atomically precise AuNCs in our system may provide a higher density of catalytic sites compared to the larger bulk gold particles used in the previously studied system, resulting in more efficient propulsion. Finally, we note that experimental environments in these studies differ significantly from ours. While the systems using bulk gold or gold nanoparticles operated in different concentrations of hydrogen peroxide (1–30%) and open aqueous environments, our system uses a closed microscope cell with 5% hydrogen peroxide as fuel, minimizing external enhancements and providing a controlled setting for evaluating intrinsic photocatalytic performance. Our results and those of Negishi also underscore the critical role of the Cr_2_O_3_ interlayer,^[^ ^36^ ^]^ which provides robust adhesion for the AuNCs while maintaining structural integrity and catalytic efficiency. In systems using gold nanoparticles on surfaces, the absence of an intermediary layer often leads to reduced adhesion stability causing detachment, leading to limited long‐term catalytic performance.^[^ ^58^ ^]^ The Cr_2_O_3_ interlayer in our system addresses this challenge, ensuring stable integration of AuNCs and sustained activity over time.

## 3 Conclusions

In conclusion, we demonstrate light activated propulsion of microswimmers co‐catalyzed by atomically precise nanoclusters. Specifically, Janus microparticles of TiO_2_ on SiO_2_ were created by physical vapor deposition. To the TiO_2_ surface, a thin layer of Cr_2_O_3_ was added to aid in the adherence and long‐term stability, of gold nanoclusters and to potentially prevent nanoclusters from sintering or agglomerating during calcination or catalysis.^[^ ^31^ ^]^ A suite of glutathione protected nanoclusters were synthesized and characterized by UV‐Vis, AFM, gel electrophoresis and EDX/STEM. These Au:SG NCs were shown, as expected, to be a mixture of cluster sizes as demonstrated by AFM and gel electrophoresis. Further, the UV‐Vis spectrum demonstrated the characteristic band structure for a mixture of atomically precise nanoclusters; yet the spectrum did not show characteristics of a gold surface plasmon resonance, demonstrating that clusters but not plasmonic particles were responsible for the observed co‐catalytic activity. A hybrid material was created by drop casting the Au:SG NCs onto the Janus TiO_2_/Cr_2_O_3_ microparticles. AuNC‐microswimmers were created post calcination, as demonstrated by STEM/EDX analysis. These particles were demonstrated to have two distinct surfaces necessary to create particles with directed motion, as characterized by STEM and EDX.

The as created AuNC‐microswimmers were shown to have light‐activated motion, from AuNC as cocatalyst, that was substantially greater than that of controls and TiO_2_/Cr_2_O_3_ microswimmers, as demonstrated by qualitative (video) and quantitative measure (mean squared displacement). It is proposed that AuNC may mechanistically enhance propulsion through a decrease in recombination due to increased charge separation with the AuNC acting as a co‐catalyst. However, the determination of the precise photochemical mechanism is beyond the scope of this study. While the purported role of AuNCs in this study is one of serving as a co‐catalyst, the potential for AuNC as a direct photocatalysis, or coupling the two mechanisms remains a potential future direction.

These results suggest that ultra small atomically precise nanoclusters, with a range of catalytic substrates and controlled size or dopant dependent optical transitions may serve in the future as ideal ultra small cocatalysts for active matter. More fundamental studies may focus on nanocluster deposition strategies, enabling concentration and location dependent targeting of ultra small nanoclusters to key areas of the microswimmer surface. Controlling both the concentration and spatial arrangement of clusters could thus control the trajectory of the microswimmer, utilizing even a single type of nanocluster with the same optical excitation mechanism. Additionally, the palette of different optically active nanoclusters, may yield the ultimate control of swimmer trajectory (e.g., by toggling different wavelengths of light); the time‐gated synchronization and bulk actuation of all microswimmers (e.g., by bulk excitation of clusters into a dark state and laser pumping them in unison out of that dark state); or strong quantum confinement similar to molecular electronic properties of atomically precise nanoclusters. Thus, these nanocluster microswimmers not only enable new fundamental physical studies, above, but enable new practical applications.^[^ ^5^, ^6^, ^7^ ^]^ For example, the varied catalytic substrates of AuNC‐microswimmers position them as promising candidates for environmental catalysis, such as the degradation of organic pollutants in aqueous systems. Additionally, their ability to harness light‐driven motility, through toggling of different wavelengths of light or harnessing different concentrations and precise localization of nanoclusters, suggests long term applications in targeted drug delivery, where light could be used to precisely control motion, over long‐time scales, in biological environments.

## 4 Experimental Section

### Microswimmer Fabrication

Fabrication of microswimmers starts with deposition of SiO_2_ microspheres onto silicon Si(100) wafers (University Wafers) by means of a Langmuir–Blodgett (LB) trough (Kibron MTX), as previously described.^[^ ^41^ ^]^ Briefly, 500 µL of 2.2 µm SiO_2_ microspheres (Bangs Laboratories) were first washed several times with nanopure water and recovered by centrifugation for ≈1 min. The aqueous solution was then replaced with 500 µL of ethanol. The particles were functionalized by addition of 250 µL of (3‐aminopropyl)triethoxysilane (APTES) (99% Sigma‐Aldrich), and reacted for 72 h rendering the particles hydrophobic. A single 100 mm diameter Si(100) wafer cut in half was submerged into the LB trough filled with deionized water (DI) water. 500 µL of the functionalized microspheres were dispersed over the surface of the water, creating a monolayer film, wherein the film was transferred by slowly removing the wafer from the bath while approximately maintaining a constant target surface pressure, which was typically in the range of π = 5–40 mN m^−1^, using the Wilhelmy plate method. The wafer was air dried. Lastly, cleaning of the microsphere monolayer was performed by placing the wafer in a Harrick PDC‐32G Basic Plasma Cleaner for 5 min wherein oxygen plasma was used to remove any remnants of the silane on the monolayer of microspheres.

The deposition of source metal titanium (Ti) pellets (Kurt J. Lesker) was carried out via electron‐beam physical vapor deposition (EBPVD) at a vacuum pressure of P ≤ 2.0 × 10^−5^ Torr. Pure Ti was deposited to a thickness of 200 nm, as controlled by the PVD instrument, at a 0° normal incidence angle, ensuring that the Ti was only deposited on the exposed hemisphere of the SiO_2_ microspheres. After deposition, the wafer was removed from the chamber and annealed in a Thermolyne 21 100 Furnace at ≈500 °C for 2 h. This annealing process converted the Ti into titanium dioxide (TiO_2_), likely forming the anatase crystalline phase known to enhance photocatalytic properties. Following annealing, the microspheres were returned to the EBPVD chamber with a new source metal of chromium oxide (Cr_2_O_3_) pellets (Kurt J. Lesker) for the deposition of a thin, ≈1 nm adhesion layer at a 0° normal incidence angle, ensuring that the Cr_2_O_3_ was only deposited on the annealed and exposed TiO_2_ hemisphere. Alternatively, a system with a thicker Cr_2_O_3_ layer (≈20 nm) was fabricated, however, we observed that the thicker layer impeded the interaction between the TiO_2,_ and the gold nanoclusters deposited in later steps.

### Synthesis and Characterization of Atomically Precise Gold Nanoclusters

The synthetic method is identical to methods reported previously for AuNC protected with glutathione ligands.^[^ ^34^, ^59^ ^]^ First, 0.25 mM (98 mg) of HAuCl_4_ (Aldrich) was added to 50 mL methanol in a 100 mL round bottom flask and was briefly sonicated to dissolve. Next, 1 mM (307 mg) of glutathione (GSH, Acros Organics) was added to the same reaction flask which was then sonicated and stirred for ≈5 min at room temperature. The mixture was then cooled to ≈0 °C in an ice bath for 30 min. An aqueous solution of 0.2 M (95 mg in 12.5 mL water) NaBH_4_ (Aldrich), kept separately in an ice bath for 30 min, was rapidly injected into the reaction flask while magnetically stirring. The reaction was stopped after an hour and the product was collected as a precipitate using centrifugation (≈3220 g for 2 min). The Au:SG nanocluster product was cleaned twice by redissolving the precipitate in water and precipitating the product using methanol. To be specific, the precipitated Au:SG product was redissolved in minimal nanopure water (≈5 mL) and then precipitated out of solution by adding 15 mL methanol. After adding methanol, the mixture was vortex stirred to dissolve any unreacted material, and the desired product was collected as a precipitate using the same centrifugation process as above. The resultant Au:SG precipitate was then dried by vacuum desiccation for 24 h, collected as a dark brown powder and stored at refrigerator temperature. For the PAGE separation of clusters, a gel of 30% acrylamide resolving and a 4% acrylamide stacking were used. The gels were run overnight using Thermo Scientific vertical electrophoresis system (P10DS) at a constant 300 V while cooling the gels at 0 °C.

The as synthesized Au:SG nanoclusters, a mixture of different sized clusters with GSH protection, were rehydrated in nanopure water (1 mL nanopure water per 10 mg AuNC powder). A more dilute solution of the Au:SG nanoclusters was also made by rehydrating the synthesized Au:SG nanoclusters in nanopure water (1 mL nanopure water per ≈1.6 mg AuNC powder) and both solutions were characterized by UV‐Vis spectroscopy (Cary 60 UV‐Vis Agilent) (Figure S2, Supporting Information, relative UV‐Vis used to more accurately compare concentrations between experiments) and atomic force microscopy (AFM). Briefly, AFM measurements were performed using an Asylum Research MFP‐3D‐Bio AFM with V16.19.220 software. Olympus AC160 probes were used to measure the samples in AC mode with the image range as reported. Nanoclusters were deposited on mica by spin coating or drop casting of 30 µL of the hydrated Au:SG NCs dilute solution (1 mL nanopure water per 1.6 mg AuNC powder) further diluted (1:99 ratio) on mica, followed by air drying. Images of nanoclusters were collected with 256 pixels x 256 lines at rates from 0.50 to 1.00 Hz per line. In addition to image collection and rendering via 2D and 3D images as shown in (Figure S3, Supporting Information), images were analyzed to determine average height of nanoclusters and reported.

### Assembly and Characterization of Hybrid AuNC‐Microswimmers

Au Nanoclusters were deposited onto the Janus particles of TiO_2_/Cr_2_O_3_ while the particles were adhered to the silicon wafer. Briefly, two aliquots of 30 µL of rehydrated Au:SG NCs (1 mL nanopure water per 10 mg or ≈1.6 mg AuNC powder, “higher” and “lower” concentration respectively) in nanopure water were sequentially drop cast from a pipette onto the silicon wafer. The microspheres were then dried in a vacuum desiccator for 24 h after each aliquot addition. For all assemblies the microswimmers were then exposed to heat treatment in a vacuum‐controlled furnace at a pressure of ≈10^−2^ torr and a temperature of ≈330 °C for 3 h to remove glutathione ligands from the Au:SG NCs, ensuring optimal interaction between the gold, Cr_2_O_3_, and TiO_2_.

The silicon wafer of TiO_2_/Cr_2_O_3_/AuNC Janus microparticles (“AuNC‐microswimmers”) and all controls were each placed into glass vials with enough 18.2 MΩ nanopure water to cover the wafer. The microswimmers were then detached from the silicon wafer surface, separated from the array of other neighboring attached microswimmers, and dispersed into the water using gentle bath sonication (SRA Digital Ultrasonic Cleaner) to create a colloidal solution.

The dispersed microswimmers were characterized by scanning transmission electron microscopy (STEM) and Energy Dispersive X‐ray (EDX) spectroscopy of Si, Ti, Cr, and Au using a ThermoScientific Talos F200i microscope operated at 200 kV. Briefly, 10 µL of dispersed microswimmers in nanopure water were drop‐cast onto a copper 300 mesh grid (Electron Microscopy Sciences) and subsequently dried in a desiccator. EDX spectroscopy was performed using specific spectral lines for accurate element identification: Si‐Kα and Si‐Kβ for silicon, Ti‐L, Ti‐Kα, and Ti‐Kβ for titanium, Cr‐L, Cr‐Kα, and Cr‐Kβ for chromium, and Au‐N, Au‐M, Au‐Lα, and Au‐Lβ for gold. These spectral lines ensured detection and differentiation of the elemental composition in the microswimmer samples.

### Characterization of AuNC‐Microswimmer Motion and Catalysis

The glass microscope slides were first cleaned using sudsy hot water with alconox for ≈2 min, followed by sequential rinsing with a copious amount of nanopure water and ethanol (squirt bottles) until the slide was not observed to contain soap, and then dried with compressed nitrogen. A final cleaning step was performed by placing the microscope slide in Harrick PDC‐32G Basic Plasma Cleaner wherein oxygen plasma was used to remove any organic remnants on the microscope slide surface for 10 min. An observation cell was constructed by cutting a square hole from a piece of double‐sided tape, which was adhered to the cleaned glass slide. A hydrophobic pen (Daido Sangyo) was used to form a barrier around the inner edge of the tape to prevent fluid from escaping the cell. A 3 µL droplet of the AuNC‐microswimmer colloidal solutions, as prepared above, were pipetted into each cell, 3 µL of freshly diluted hydrogen peroxide was then added and the cell was sealed with a clean glass cover slide. The final concentration of hydrogen peroxide in all experiments was held constant at 5% (v/v).

The microswimmers were excited with UV light (λ = 365 nm) and green light (λ = 562 nm), separately, at the maximum possible intensity of the microscope (Nikon Eclipse Ti2‐U Microscope), ≈1.6 µW cm^−2^. Videos of the particles in action were recorded at ≈15 fps using a Thorlabs DCU223 CCD camera, and the motion was analyzed using the MTrack2 plugin in ImageJ to obtain 2D trajectories of the swimmers. The minimum number of particles measured was 75, and the maximum was 90 for each experiment. Each particle was tracked for a minimum of 10 s, resulting in ≈150 frames that were used to calculate individual average speeds. Microswimmer average speed and frequency distributions were calculated using MATLAB from the trajectories obtained through particle tracking.

### Statistical Analysis

All experimental data were processed to ensure accuracy and reproducibility. Video pre‐processing steps were employed to ensure consistent trajectory extraction. Pre‐processing steps are as follows. Videos were converted to binary images in ImageJ, where the background and non‐microswimmer regions were set to white while microswimmers remained black. This facilitated precise particle tracking using the MTrack2 plugin, which was used to obtain time‐resolved positional data and reconstruct individual microswimmer trajectories. No data were excluded; all observed microswimmers were included in the analysis.

Data are presented as mean ± standard error of the mean (SEM) for MSD analysis (Figure 4a) and mean ± standard deviation (SD) for speed histograms (Figure 4b). A minimum of *n* = 75 microswimmers per condition was analyzed to ensure statistical significance.

To determine significant differences between control and experimental groups for speed analysis, a two‐sided Mann‐Whitney U test was used due to the non‐Gaussian distribution of velocity data. The significance threshold was set at p < 0.001.

All data analysis, statistical comparisons, and visualization were performed using MATLAB, including ImageJ for trajectory extraction, MATLAB for velocity calculations and statistical testing, and MATLAB‐generated plots for visualization.

Supporting information contains further analysis of Au:SG NCs (UV‐vis spectra, gel separation, and AFM), additional characterization of AuNC‐microswimmers and their controls, and original data (video) that contributed to the still frames within Figure 1 and Figure 3.

## Conflict of Interest

The authors declare no conflict of interest.

## Supporting information



Supporting Information

Supporting Information

Supporting Information

Supporting Information

Supporting Information

Supporting Information

## Data Availability

The data that support the findings of this study are available from the corresponding author upon reasonable request.
