# Supplementary material for: Atomically Precise Nanoclusters as Co‐Catalysts for Light‐Activated Microswimmer Motility
Source: Small. 2025 May 16;21(25):2411517. doi: 10.1002/smll.202411517 (PMC12199103; doi:10.1002/smll.202411517)
Supplement: Supplementary file 1 — Supporting Information [file SMLL-21-2411517-s004.docx]

**Supplemental Information**

**Atomically Precise Nanoclusters as Co-Catalysts for Light-Activated Microswimmer Motility**

*John Castañeda^1^, Blake Rogers^1^, Ysaris Sosa^1^, Jorge A. Muñoz^1^, Badri Bhattarai^1^, Ashley M. Martinez^1^, M. Lisa Phipps^2^, Demosthenes P. Morales^2^, Matthew N. Montoya Rush^3^, Miguel Jose Yacaman^1^, Gabriel A. Montaño^1^*, John G. Gibbs^1^, Jennifer S. Martinez^1,2^**

1. Department of Applied Physics and Materials Science and Center for Materials Interfaces in Research and Applications (¡MIRA!)

Northern Arizona University

1900 S Knoles Dr

Flagstaff AZ, 86011, United States

2. Center for Integrated Nanotechnologies

Los Alamos National Laboratory

P.O. Box 1663

Los Alamos, NM, 87545, United States

3. Washington University in St. Louis School of Medicine, Department of Surgery

1402 S Grand Blvd

St. Louis, MO, 63104, United States

E-mail: [jenm@lanl.gov](mailto:jenm@lanl.gov) (JSM) and [gabriel.montano@nau.edu](mailto:gabriel.montano@nau.edu) (GAM)

**Contents:**

Video of AuNC-microswimmers and their controls, from which still images were extracted for Figure 1 and Figure 3, are uploaded on the Journals website.

Figure S1. Characterization of microswimmers with a thick Cr_2_O_3_ (20 nm) layer.

Figure S2. Gel electrophoresis of Au:SG NCs and UV-Vis comparison of two concentrations of Au:SG NCs used in creation of two different AuNC-microswimmer populations.

Figure S3. AFM images of as synthesized Au:SG NCs (with ligands) used to determine nanocluster size.

Figure S4. The mean squared displacement (MSD) for AuNC-microswimmers and control TiO_2_/Cr_2_O_3_ microswimmers, with and without light excitation.

Figure S5. Comparative analysis of the motility of AuNC-microswimmers and control TiO_2_/Cr_2_O_3_ microswimmers with and without UV (365 nm) and visible light (532 nm) excitation.

Figure S6. Reactivity of AuNC-microswimmers with a lower concentration of deposited AuNCs.

***
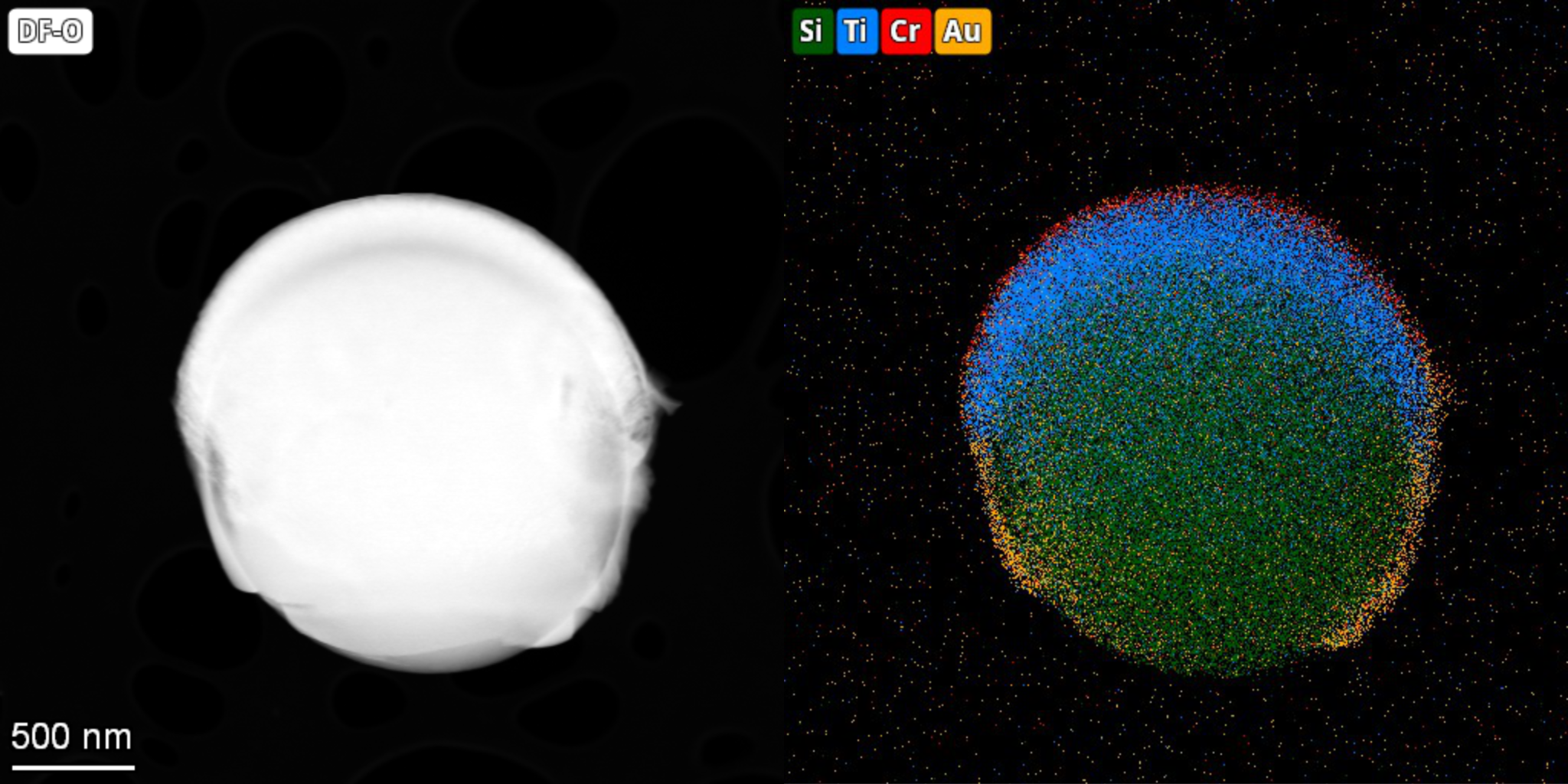
*Figure S1. Characterization of microswimmers with a thick Cr_2_O_3_ (20 nm) layer.** Scanning transmission electron microscopy (STEM) images show the microswimmer structure, and their elemental distribution of Si (green), Ti (blue), Cr (red) and Au (yellow) using Energy Dispersive X-ray (EDX) spectroscopy. While this image shows a microswimmer with the thick Cr_2_O_3_ and an AuNC layer, this and a similar control (without AuNC) did not demonstrate the photocatalytic activity expected for TiO_2_ microswimmers (e.g. it did not exhibit the reactivity observed in Figures 3, 4, or Figures S4, S6); instead only Brownian motion was observed.

***
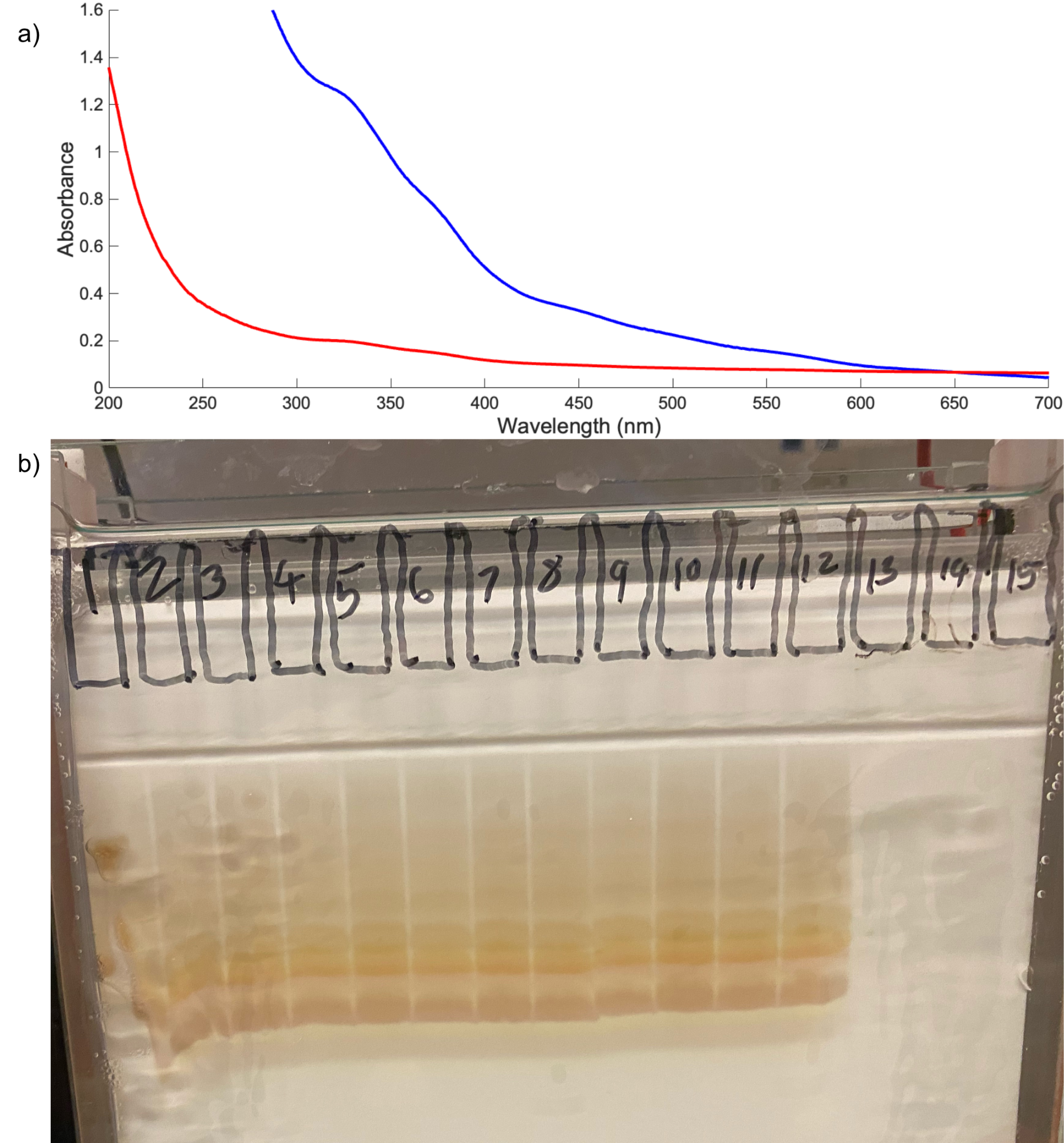
***

**Figure S2.** **Gel electrophoresis of Au:SG NCs and UV-Vis comparison of two concentrations of Au:SG NCs used in creation of two different AuNC-microswimmer populations.** a) UV-Vis spectra of glutathione protected gold nanoclusters (Au:SG NCs) at two different concentrations (higher concentration, blue; lower concentration, red). The relative difference in concentration was then utilized to create two different AuNC-microswimmer populations each with differing quantities of AuNC. b) Au:SG NC sizes as resolved by polyacrylamide gel electrophoresis (30% gel and 4% stack). This synthetic method is known to produce a mixture of Au:SG NCs from 10 to 39 gold atoms, derived from the nine bands that have been isolated using polyacrylamide gel electrophoresis (PAGE), here too we observe a mixture of Au:SG NCs consistent with AFM and the UV-Vis.^[1,2]^

**
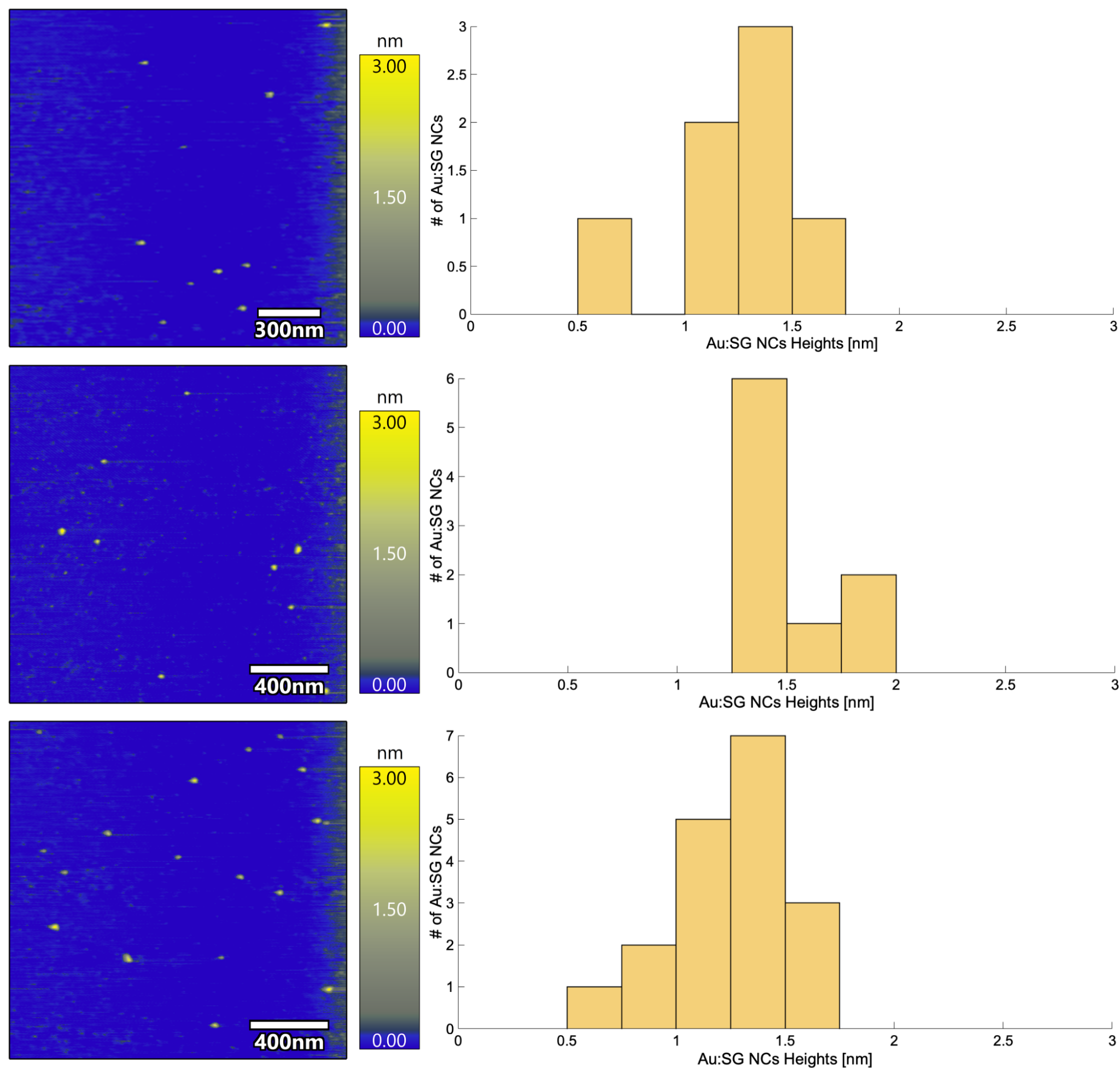
**

**Figure S3.** **AFM images of as synthesized Au:SG NCs (with ligands) used to determine nanocluster size.** The 2D images (left) of Au:SG NCs on mica are flattened 2D images that were used to determine the z-height (size) of Au:SG NCs. The heatmap depicts the height of the nanoclusters, while the histogram on the right plots the average height distribution of the nanoclusters. AFM samples were prepared by depositing 30 microliters of the Au:SG rehydrated solution on atomically flat mica at concentrations that rendered largely individual nanoclusters at a density capable of visualizing enough NCs for statistical size distribution analysis. AC-mode AFM was used to analyze Au:SG NCs prior to calcining by measuring the Z-height of NC’s deposited on mica. As can be observed, NCs were found to have an average height between 0.5-2 nm consistent with UV-Vis for pre-calcined AuNCs. Z-height analysis is the most effective means of analyzing particles that are smaller than the radius of the AFM tip (~7 nm); otherwise, the larger radius AFM tip causes “tip broadening” in measuring lateral dimensions and can be clearly observed by the cone-shaped appearance in the 3D rendered image of Figure 2b.^[3]^

***
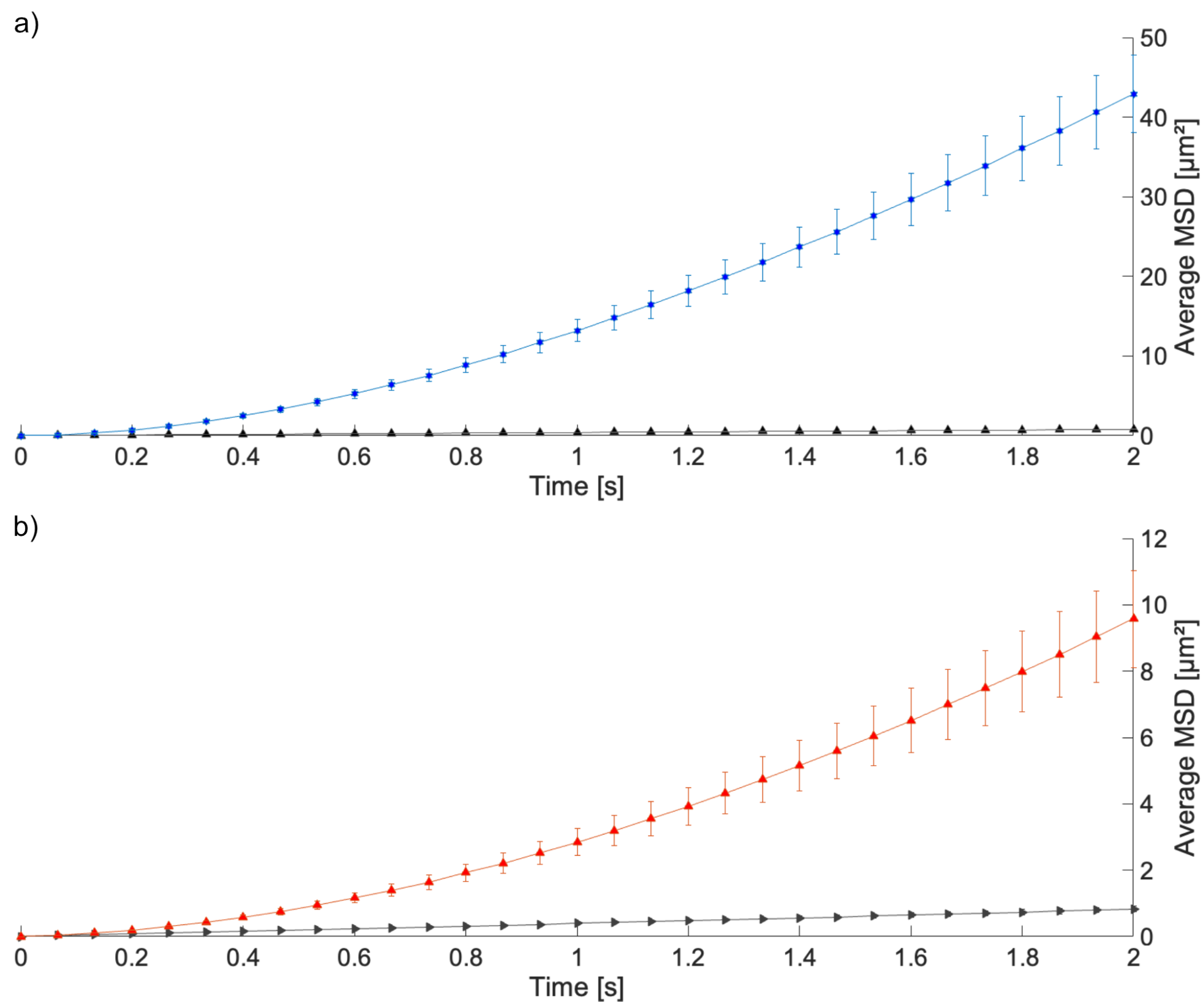
***

**Figure S4. The average mean square displacement (MSD), over two seconds, for AuNC-microswimmers and control TiO_2_/Cr_2_O_3_ microswimmers, with and without light excitation.** (a) MSD for AuNC-microswimmers with (blue) and without UV light excitation (black). (b) MSD for TiO_2_/Cr_2_O_3_ microswimmers with (red) and without UV light excitation (black), notice the difference in scale for the Y axis between panels a and b. For both microswimmers, the motility is observed to be light dependent, and likely due to the redox reactions indicated within the manuscript. It is also observed that AuNC-microswimmers have quantitatively enhanced reactivity resulting from the gold nanoclusters. A linear curve fitting of Equation 2 for diffusion of the microswimmers without UV light resulted in identical diffusion coefficients of ~0.10 μm²/s.

**
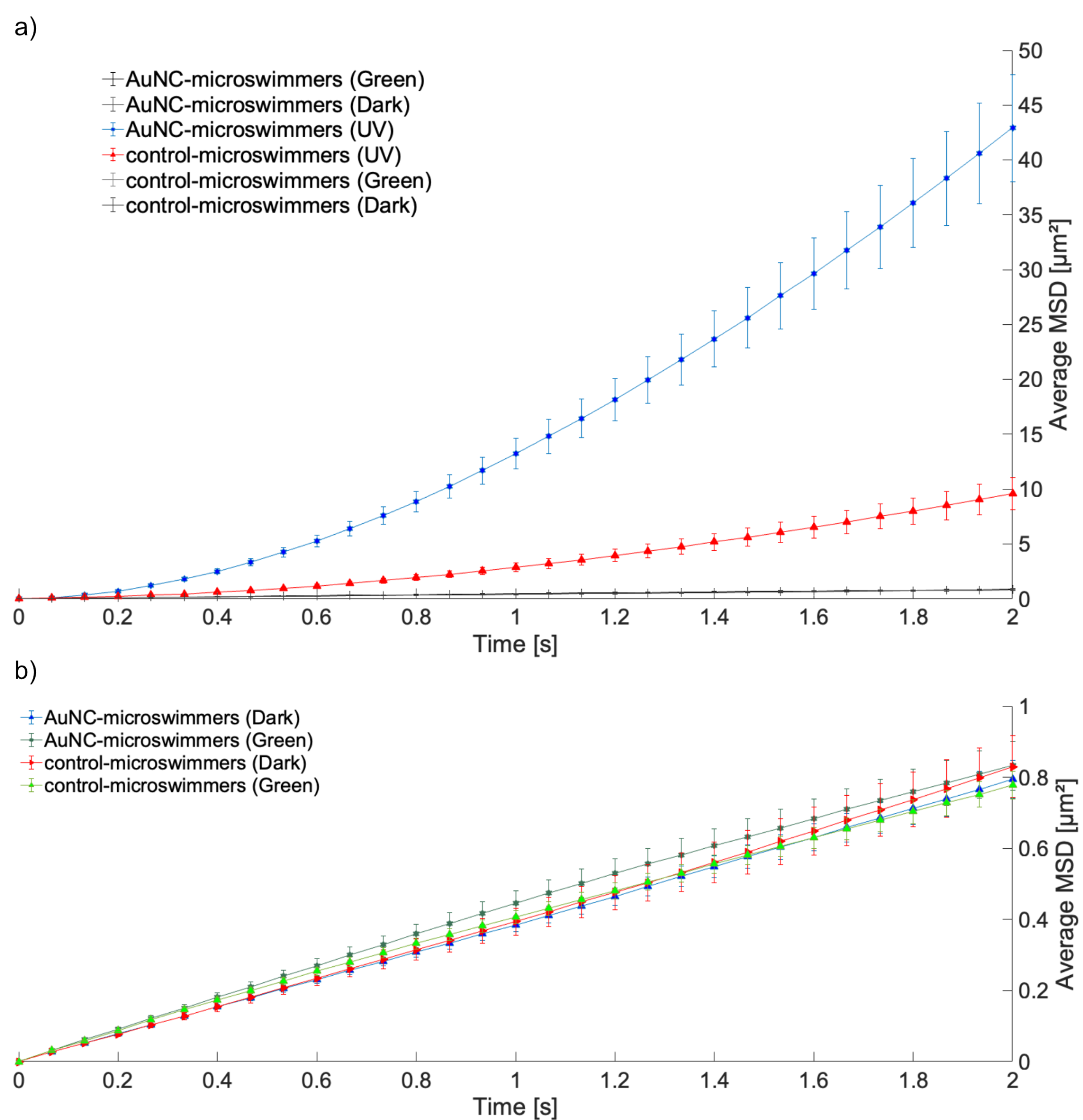
**

**Figure S5.** **Comparative analysis of the motility of AuNC-microswimmers and control TiO_2_/Cr_2_O_3_ microswimmers with and without UV (365 nm) and visible light (532 nm) excitation.** (a) The plot shows the mean squared displacement (MSD) for the AuNC-microswimmers and the control TiO_2_/Cr_2_O_3_ microswimmers with and without UV and visible light excitation. (b) Magnification of MSD plots with and without visible light excitation for both the AuNC-microswimmers and the control TiO_2_/Cr_2_O_3_ microswimmers. The diffusivity constants determined by a linear fit of Equation 2 to the MSD curves were ~0.10 μm²/s for the dark and green light conditions. This indicates that visible light-activated catalysis does not occur with the current AuNC conditions. Further studies will be required to couple the optical properties of the AuNC to the TiO_2_ microparticles to broaden the excitation range for propulsion.

***
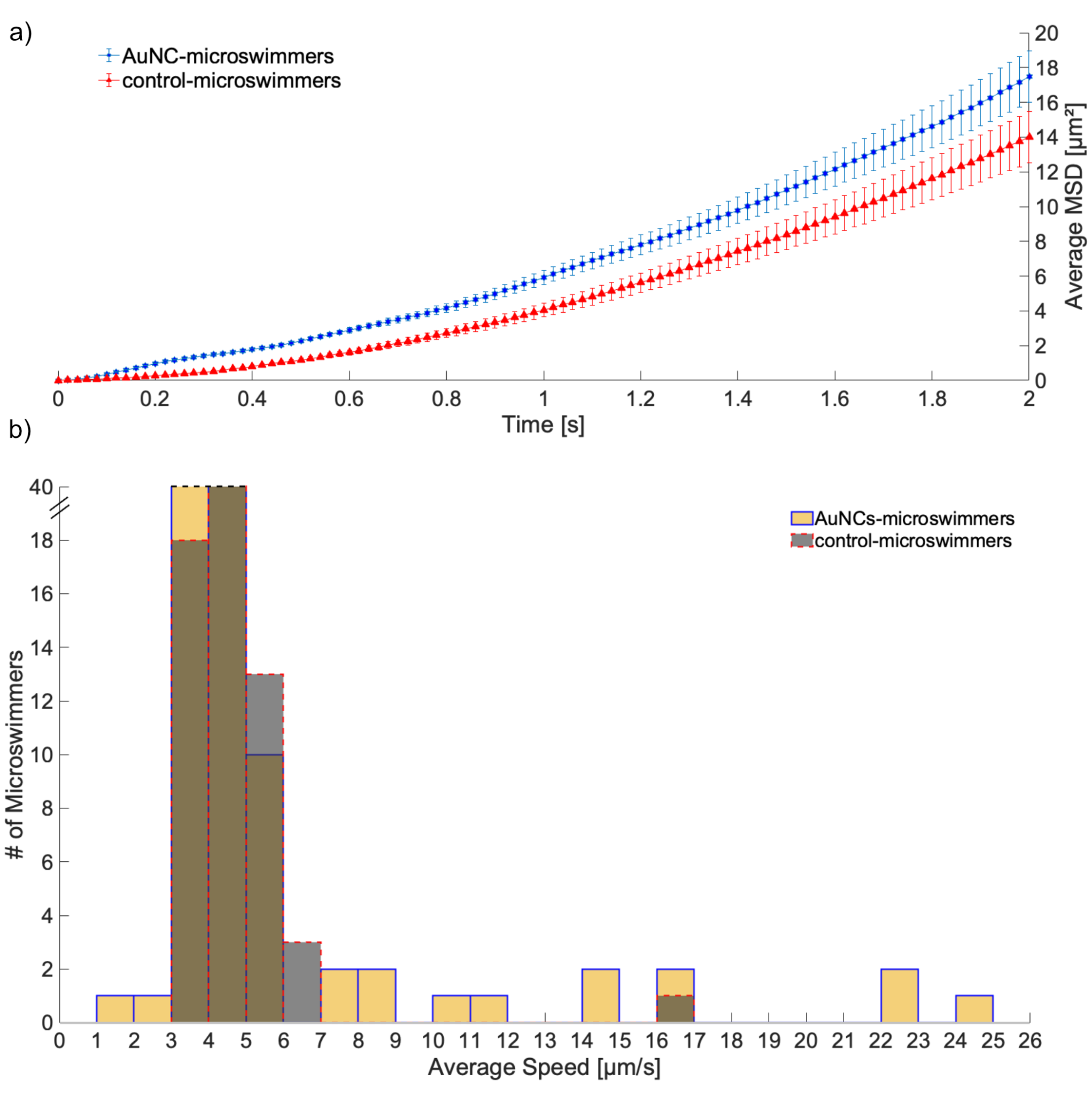
***

**Figure S6. Reactivity of AuNC-microswimmers with a lower concentration of deposited AuNCs.** (a) The mean squared displacement (MSD) for the AuNC-microswimmers (blue) and the control TiO_2_/Cr_2_O_3_ microswimmers (red). As demonstrated in Figure S2, we find the concentration of AuNC within this experiment to be ~6.25-fold less than that of Figures 3 and 4; while the reduction in MSD scaling is approximately 3-fold less. Fitting Equation 4 to the MSD curves determined that the mean velocities were similar with a mean velocity of 1.66 ± 0.07 µm/s for the low AuNC-microswimmers and 1.74 ± 0.06 µm/s for the control TiO_2_/Cr_2_O_3_ microswimmers. However, the estimated diffusion coefficient of the TiO_2_/Cr_2_O_3_ microswimmers was 3-fold lower than the AuNC-microswimmers measuring 0.250 ± 0.005 μm²/s and 0.79 ± 0.008 μm²/s, respectively. This may indicate a non-uniform distribution of nanoclusters on the surface of the microswimmers resulting in asymmetric propulsion of the particles. (b) The average speed frequency distribution for the AuNC-microswimmers (yellow) compared to the control TiO_2_/Cr_2_O_3_ microswimmers (gray). The control microswimmers exhibit a clear peak in average speed, centered over a spread in speed between ~3 and 6 µm/s for this experiment. Whereas a wide distribution of speeds, ranging from approximately ~1 μm/s to 24 μm/s was observed for the AuNC-microswimmers.

**References for Supplemental Information:**

[1] Y. Negishi, K. Nobusada, T. Tsukuda, *J. Am. Chem. Soc.* **2005**, *127*, 5261.

[2] S. Li, X. Du, Z. Liu, Y. Li, Y. Shao, R. Jin, *Precis. Chem.* **2023**, *1*, 14.

[3] I. Misumi, K. Sugawara, K. Takahata, K. Takahashi, K. Ehara, *Precis. Eng.* **2018**, *51*, 691.
